# Recent Advances in Heterologous Synthesis Paving Way for Future Green-Modular Bioindustries: A Review With Special Reference to Isoflavonoids

**DOI:** 10.3389/fbioe.2021.673270

**Published:** 2021-07-01

**Authors:** Moon Sajid, Shane Ramsay Stone, Parwinder Kaur

**Affiliations:** UWA School of Agriculture and Environment, University of Western Australia, Perth, WA, Australia

**Keywords:** synthetic biology, heterologous synthesis, green bioindustries, isoflavonoids, plant secondary metabolites

## Abstract

Isoflavonoids are well-known plant secondary metabolites that have gained importance in recent time due to their multiple nutraceutical and pharmaceutical applications. In plants, isoflavonoids play a role in plant defense and can confer the host plant a competitive advantage to survive and flourish under environmental challenges. In animals, isoflavonoids have been found to interact with multiple signaling pathways and have demonstrated estrogenic, antioxidant and anti-oncologic activities *in vivo*. The activity of isoflavonoids in the estrogen pathways is such that the class has also been collectively called phytoestrogens. Over 2,400 isoflavonoids, predominantly from legumes, have been identified so far. The biosynthetic pathways of several key isoflavonoids have been established, and the genes and regulatory components involved in the biosynthesis have been characterized. The biosynthesis and accumulation of isoflavonoids in plants are regulated by multiple complex environmental and genetic factors and interactions. Due to this complexity of secondary metabolism regulation, the export and engineering of isoflavonoid biosynthetic pathways into non-endogenous plants are difficult, and instead, the microorganisms *Saccharomyces cerevisiae* and *Escherichia coli* have been adapted and engineered for heterologous isoflavonoid synthesis. However, the current *ex-planta* production approaches have been limited due to slow enzyme kinetics and traditionally laborious genetic engineering methods and require further optimization and development to address the required titers, reaction rates and yield for commercial application. With recent progress in metabolic engineering and the availability of advanced synthetic biology tools, it is envisaged that highly efficient heterologous hosts will soon be engineered to fulfill the growing market demand.

## Introduction

Living organisms like plants, fungi and unicellular prokaryotes and eukaryotes produce a myriad of chemicals, broadly classified as primary metabolites and secondary metabolites. Primary metabolites are fundamental compounds of life as they are involved in vital cellular processes such as respiration and photosynthesis. Secondary metabolites are a specialized class of chemicals, and they are usually produced under specific conditions and give producing organisms an additional advantage to survive, compete or attack other organisms (Nabavi et al., 2020). Secondary metabolites are usually divided into three classes (alkaloids, terpenoids, and phenylpropanoids) based on their chemical structure and precursor primary metabolite. The phenylpropanoid is the largest class of plant secondary metabolites, which play an important role in plant growth and development (Vogt, 2010). The phenylpropanoid pathway has different branches that lead to different groups of compounds including chalcones, flavanones, isoflavonoids, and anthocyanins (Novelli et al., 2019). The structure and function of most of these groups have been well researched and documented in the literature.

**FIGURE 1:**
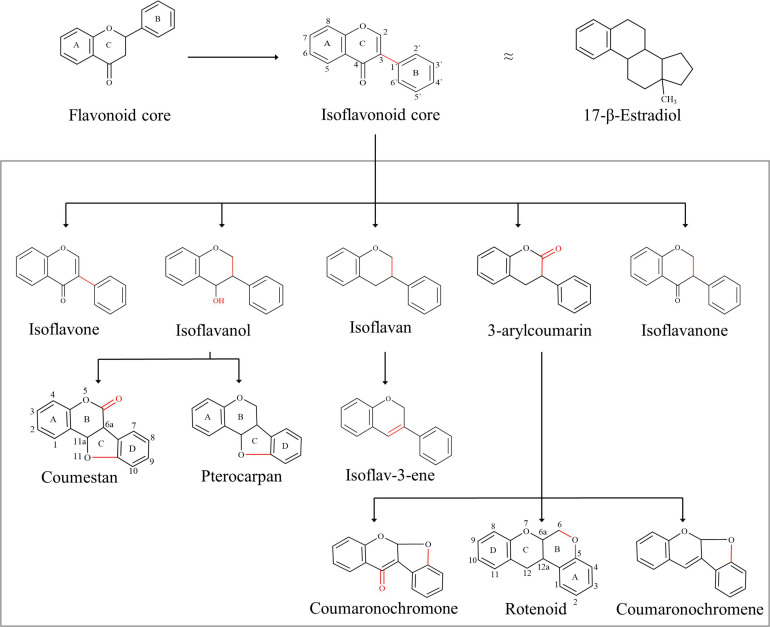
Basic skeleton of isoflavonoids: isoflavonoids are structurally different to flavonoids, with the B-ring migration from position 2 to 3, which in turn leads to the structural similarities to estrogen, e.g., 17β-estradiol. Isoflavonoid diversity is regulated by simple functional additions such as hydroxyl, which in turn can generate additional rings into the backbone, e.g., pterocarpan and coumestan. The addition of ketones can also generate additional rings, for example, rotenoid and coumaronochromone.

The isoflavonoids are a large group of plant secondary metabolites and possess a 3-phenylchroman skeleton, which is biogenetically derived from the 2-phenylchroman skeleton of the parent flavonoid (Figure 1). Isoflavonoids are predominantly present in Papilionoideae, a subfamily of Leguminosae (Dixon and Sumner, 2003). More than 2,400 isoflavonoids from over 300 plants have been identified so far (Veitch, 2007, 2009, 2013; Al-Maharik, 2019). Isoflavonoids play multiple roles in host plant, and their role in plant defense and plant–rhizobia relationships is the most significant (Larose et al., 2002). Due to their significance for the host plant, biosynthetic pathways involved in the synthesis and accumulation of many isoflavonoids have been explored. Following that, several attempts have been made to increase the content of isoflavonoids in endogenous as well as in related plants. However, due to the complexity of plant secondary metabolism, no significant improvement has been achieved.

Isoflavonoids have played a distinctive role in the history of disease prevalence across continents. It is believed that the difference in the prevalence of cancer across continents is linked with the preference for soy foods (Ko, 2014). Soybean products are rich in organic chemicals that are structurally similar to 17β-estradiol, a human sex hormone (Figure 1). Due to this structural similarity, isoflavonoids play an important role in cellular signaling pathways and control multiple functions in humans and are commonly known as phytoestrogens (Prasad et al., 2010). Together with this, isoflavonoids are commonly used in cosmetics, nutraceuticals, pharmaceuticals, food, and beverage industry; however, the pharmaceutical sector holds the largest share in the market due to potential applications of isoflavonoids in chronic and cardiovascular diseases. The market size of isoflavonoids was over US$ 13.5 billion in 2018 and is estimated to reach US$ 30 billion by 2025 (Ahuja and Mamtani, 2019). Isoflavonoids are presently extracted from plants; however, alternative production platforms are also being explored for their sustainable production to maintain a constant supply in the growing market.

Due to a range of potential applications, the demand for isoflavonoids is growing in recent times. However, the issue of traditional extraction and low yield in plants along with recent climate change and competition to use cultivatable land have questioned their availability for the general public. Therefore, the present paper is aimed to discuss the biosynthetic pathways and potential applications of isoflavonoids (as a subclass of flavonoids) generally but specifically about seven key isoflavonoids: daidzein, formononetin, pisatin, medicarpin, coumestrol, genistein, and biochanin-A with a special focus on their biosynthesis in heterologous hosts.

## Industrial Applications of Isoflavonoids

Isoflavonoids are commonly present in low amounts in seeds and roots of the Leguminosae/Fabaceae family including several commonly consumed plants like barley, broccoli, cauliflower, fava beans, lupine, kudzu, and soy (Prasad et al., 2010; Table 1). Traces of isoflavonoids are also present in red wine and in other plants like alfalfa, red clover and linseed (Pilsakova et al., 2010). Quite interestingly, isoflavonoids have also been identified from at least 59 non-leguminous plant families (i.e., Iridaceae, Rosaceae, and Liliaceae), as it is commonly believed that isoflavonoids’ biosynthetic machinery is not widely distributed in plant families except legumes (Lapčík, 2007).

**TABLE 1:** Concentration of key isoflavonoids in common food and forage legumes.

Botanical name (common name)	Daidzein (μg/g)	Genistein (μg/g)	FMN (μg/g)	Biochanin-A (μg/g)	CMS (μg/g)	References
*Arachis hypogaea*(peanut)	0.49	0.82	0.06	0.06	0	Mazur et al., 1998
*Cajanus cajan*(pigeon pea)	0.14	7.37	318 (leaf)	405 (leaf)	tr	Mazur et al., 1998; Wei et al., 2013
*Cicer arietinum*(chick pea)	0.11	0.01 (flour)	0.02 (flour)	0.78 (flour)	0.05	Mazur, 1998; Megías et al., 2016
*Glycine max*(soybean)	327^a^	363^a^	0.40^b^	0.15^b^	1.85^b^	Mazur et al., 1998; Cho et al., 2020
*Phaseolus vulgaris*(kidney beans)	62^c^	77^c^	0.04	0.26	0.02	Mazur et al., 1998; de Lima et al., 2014
*Pisum sativum*(split peas)	0.07	0.22	0.04	0.05	tr	Mazur et al., 1998
*Vicia faba*(fava bean)	51 (stem)	0.48 (stem)	0.06	tr	0	Mazur et al., 1998; Fuentes-Herrera et al., 2020
*Vigna mungo*(Urd bean)	0.30	0.60	0	0.81	0.09	Mazur et al., 1998
*Vigna radiata*(mung bean)	26.30 (sprouts)	15.70 (sprouts)	0.07	0.14	tr	Mazur et al., 1998; Silva et al., 2013
*Vigna unguiculata*(cowpea)	0.30	0.55	0	0	tr	Mazur et al., 1998

*Isoflavonoid content was tested in seeds unless specified. FMN, formononetin; CMS, coumestrol; tr, present in trace amounts. ^a^Daepung no. 2 variety. ^b^Santa Rosa variety. ^c^Soja variety.*

### Role in Plants

With increasing climate and environmental pressures, the potential utilization of isoflavonoids *in planta* to enhance plant resistance against herbivore insects and to improve the interactions of the plant with the rhizobiome has resulted in increased interest and research (Dillon et al., 2017).

**FIGURE 2:**
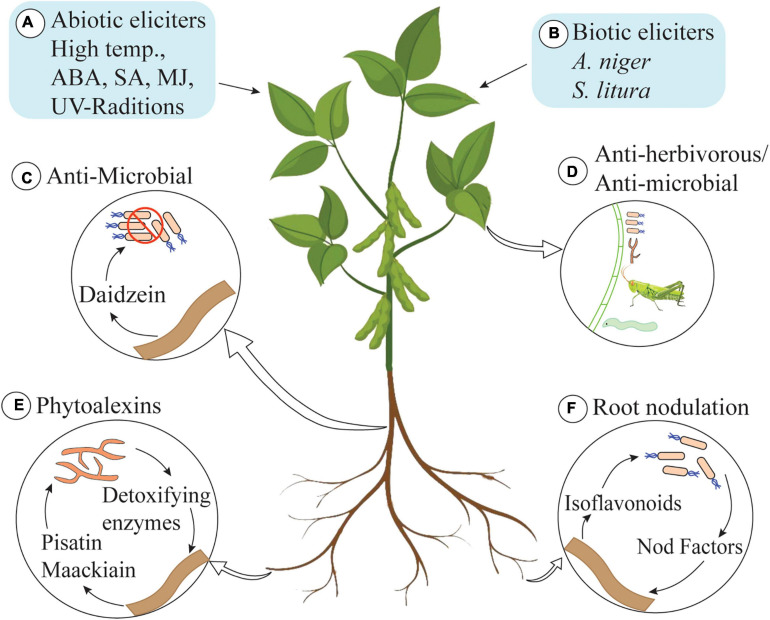
Role of isoflavonoids in plants. **(A)** Abiotic elicitors: high temperature, abscisic acid (ABA), salicylic acid (SA), methyl jasmonate (MJ), and UV-radiations. **(B)** Biotic elicitors: *Aspergillus niger* and *Spodoptera litura* trigger isoflavonoid biosynthesis in plants. **(C)** Daidzein secreted by roots kills bacteria in rhizosphere and shapes the rhizosphere communities. **(D)** Isoflavonoids also possess anti-herbivorous and antimicrobial activities. **(E)** Pisatin and maachiain kill pathogenic fungi in rhizosphere (some fungi produce detoxifying enzymes to degrade pisatin and maachiain to cause infection in roots of host plant). **(F)** Some isoflavonoids induce nodulation genes in receptive bacterial species and play an important role in root-nodule formation.

Isoflavonoids produce a spectrum of benefits for the host plant (Figure 2). Isoflavonoids play an important role in plant defense, as they possess a range of antimicrobial activities (commonly analyzed *in vitro*) (Dixon, 1999). They are famous as plant defensive chemicals and are active against vertebrates, molluscs, herbivorous insects, and microorganisms (Dakora and Phillips, 1996; Nwachukwu et al., 2013). For example, the well-known isoflavonoid pterocarpans, maackiain, and pisatin play an important role as phytoalexins in the interaction between *Nectria haematococca* and the host plant *Pisum sativum* (garden pea) (Wasmann and VanEtten, 1996; Enkerli et al., 1998). Both of these pterocarpans are targets of fungal virulence factors and detoxification enzymes, which indicates their importance for the host plant. Recently, Dillon and colleagues have shown that UV-B-induced accumulation of genistein enhances resistance of field-grown soybean plants against *Anticarsia gemmatalis* neonates (Dillon et al., 2017). A 30% reduction in survival and 45% reduction in mass gain of larvae was documented, and the authors have concluded that UV-B-induced accumulation of isoflavonoids increases the resistance of plants against *A. gemmatalis* (Dillon et al., 2017). An overview of UV-B-based induction of isoflavonoids is described in section “Regulation of Isoflavonoid Biosynthesis in Plants.”

Isoflavonoids are not only active inside the cell but also play a beneficial role in the rhizosphere. The role of isoflavonoids in the induction of nodulation genes and as allelopathic agents has also been documented (Dixon, 1999). Daidzein, secreted by soybean roots, acts as a signaling molecule for nodulation and alters the structure and functioning of rhizosphere communities (Okutani et al., 2020). In addition to this, isoflavonoids play a role in the induction of transcription of genes involved in the production of the Nod factor. These Nod factors are rhizobial signaling molecules that make plants receptive to symbiotic root infection. Some isoflavonoids are very specific and only induce the production of Nod factors in compatible hosts, thus playing an important role in host selection (Aoki et al., 2000). Isoflavonoids are also involved in developing mutualistic interaction with compatible fungal species. The role of isoflavonoid in initiating the spore germination, hyphal growth and root colonization as well as the formation of arbuscule inside the root cell has been documented (Larose et al., 2002).

### Role in Human Health

**FIGURE 3:**
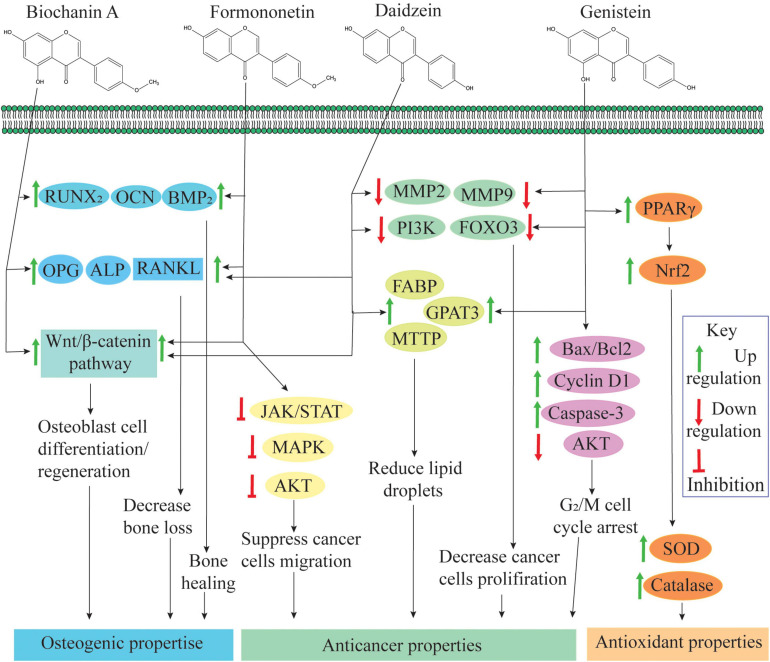
Role of isoflavonoids in human health: biochanin-A and formononetin are involved in bone healing and regeneration effects by upregulating runt-related transcription factor 2 (RUNX2), osteocalcin (OCN) and bone morphogenetic protein 2 (BMP2) expression at the injury site (Singh et al., 2017). Biochanin-A, formononetin, and daidzein also regulate osteoprotegerin (OPG), alkaline phosphatase (ALP), and receptor activator of nuclear factor κβ ligand (RANKL) expression; and these compounds are actively involved in osteogenic activities (Zakłos-Szyda et al., 2020). Additionally, an isoflavone mixture (biochanin-A, formononetin, and daidzein) promotes osteoblast cell differentiation and proliferation through the activation of the Wnt/β-catenin pathway (Chen et al., 2018). Additionally, formononetin inactivates signaling pathways, namely, Janus kinase/signal transducers, and activators of transcription (JAK/STAT) pathway, protein kinase B (PKB or AKT) pathway and mitogen-activated protein kinase (extracellular signal regulated kinase 1/2) [MAPK (ERK1/2)] pathways and suppresses cell migration, invasion and angiogenesis (Qi et al., 2016; Park et al., 2018). Daidzein and genistein can inhibit expression of matrix metalloproteinase-2/9 (MMP2/9), phosphatidylinositol-3-kinase (PI3K) and forkhead box O-3 (FOXO3) and reduce the cancer cell proliferation. Through activation of fatty acid-binding protein (FABP), glycerol-3-phosphate acyltransferase 3 (GPAT3) and microsomal triglyceride transfer protein (MTTP), genistein and daidzein reduce lipid droplet accumulation. Both of these situations induce apoptosis in cancer cells (as reviewed by Hsiao et al., 2020). Genistein activates the expression of Bcl-2-associated X-protein (Bax/Bcl-2), cyclin D1 and caspase-3 pathways and suppressed PI3K/AKT phosphorylation, which results in genistein-induced G2/M cell cycle arrest (Shafiee et al., 2016). Genistein activates peroxisome proliferator-activated receptor gamma (PPARγ) and enhances expression of superoxide dismutase (SOD) and catalase via Nrf2 activation (Chan et al., 2018).

Several epidemiological studies have shown that an isoflavonoid-rich diet is associated with a low risk of chronic diseases like menopausal, diabetes, cancer, and cardiovascular diseases (Kozłowska and Szostak-Wȩgierek, 2017). Due to chemical similarity with 17β-estradiol, isoflavonoids can bind with estrogen receptors (ERs) such as ER-α and ER-β (Chen et al., 2018). Due to this affinity, isoflavonoids interfere with cellular signaling mechanisms and play an important role in cellular growth and protection (Figure 3). Isoflavonoid aglycones (without glucose) are sometimes biologically more active and available than glycones, as glucose moiety has a strong effect on their function and absorbance in the human gastrointestinal tract (Lee et al., 2018). For example, in its un-glycosylated form, the affinity of genistein is comparable with that of 17β-estradiol, but in its glycosylated forms, its affinity is up to 100–500 times less (Kuiper et al., 1997; Breinholt and Larsen, 1998).

The application of isoflavonoids in human health is a diverse topic, which is not in the scope of the present review. Therefore, in the following paragraphs, a short and precise overview of their health applications has been covered. Interested readers are request to consult recent review papers for further details (Zaheer and Humayoun Akhtar, 2017; Das et al., 2020; Hu et al., 2020; Liu et al., 2020).

#### Estrogenic Properties

The applications of isoflavonoids as phytoestrogens are one of the most exciting areas of interest in clinical research and nutrition. The declining level of estrogen hormone in aging women is a main cause of osteoporosis, and soy isoflavonoids can substitute the natural estrogen and control bone loss (Chen et al., 2018; Zakłos-Szyda et al., 2020). A meta-analysis has shown that soy isoflavonoid intake for 6 months has a beneficial effect on bone mineral density (BMD), especially on the lumbar spine (Wei et al., 2012). That is why the prevalence of osteoporosis is low in the Chinese and Japanese populations as compared with that in European and Americans. The beneficial role of isoflavonoids is explained due to their molecular similarity with natural estrogen and binding affinity with ERs, especially with ER-β. In addition to this, isoflavonoids intake has positive effects on learning and memory expression, and isoflavonoids are also involved in controlling hot flashes in menopause women (Henderson et al., 2000; Li et al., 2015). Several isoflavonoids including genistein and daidzein as well their derivative have been identified and characterized for their beneficial estrogenic properties.

#### Antioxidant Properties

The role of isoflavonoids as antioxidants is well established; and it is sometimes believed that the antioxidant potential of isoflavonoids is comparable with that of the well-known antioxidant vitamin E (Djuric et al., 2001). Antioxidant properties are achieved either by the regulation of gene expression of antioxidant enzymes, for example, catalase, or by inhibiting the secondary oxidant production like hydrogen peroxide (Mortensen et al., 2009). Genistein and daidzein are well-known metal chelator and radical scavenger; and due to the presence of three hydroxyl groups, the former is better than the latter (Han et al., 2009). Genistein increases the production of superoxide dismutase (SOD), which scavenges free radicals (Kuriyama et al., 2013). Conversion of daidzein to equol is an important physiological phenomenon, as equol has 100-fold higher ER binding affinity and a greater antioxidant ability than daidzein (Zaheer and Humayoun Akhtar, 2017). Isoflavonoids support disease prevention like type 2 diabetes and health maintenance by repressing the oxidative stress (Umeno et al., 2016). However, it is largely unclear to which extent isoflavonoids mediate antioxidant activities *in vivo*, which is difficult to access, and their antioxidant activities are mostly accessed *in vitro*.

#### Anticancer Properties

Isoflavones are also well-known for their anticancer potential. Their role in the increase of prostacyclin level, activation of the endothelial level of nitric oxide synthase, inhibition of cell proliferation and DNA synthesis, relaxation of vessels and reduction of plaque, and maintenance of circulatory systems has also been documented (Cano et al., 2010; Tay et al., 2019). Isoflavonoids also interact with epigenetic modifications and are involved in the hypermethylation of tumor suppressor genes, and the underlying mechanism of methylation and acetylation of histones in breast cancer cell lines has also been revealed (Dagdemir et al., 2013).

Genistein, daidzein, formononetin, and, to some extent, coumestrol are well-studied isoflavonoids due to their anticancer potential. Genistein is a strong anticancer candidate isoflavonoid compound with a 50% inhibitory concentration (IC_50_) value of 37.5 μM against human topoisomerase II (Mizushina et al., 2013). Genistein is known to inhibit protein tyrosine kinase (PTK) and DNA topoisomerases I and II and, therefore, affects a range of cellular activities specifically in carcinogenesis and other neurodegenerative diseases (Kuriyama et al., 2013). Genistein and daidzein can induce cell cycle arrest at S, G_2_/M and G_1_ stages in various cancer cells (Bossard et al., 2012; Adjakly et al., 2013; Shafiee et al., 2016). In a recent study, the authors have concluded that genistein and daidzein can play an important role in cancer cell metastasis, tumorigenesis and stem-like properties, and they have potential as alternative therapies for ovarian cancer patients (Chan et al., 2018).

The IC_50_ value of formononetin ranges from 10 to 300 μM when tested against various cancer cell lines. Formononetin is also able to efficiently inhibit tumor growth *in vivo*, and it is effective against many types of tumors including breast, bone, colon, nasopharyngeal and multiple myeloma cells (Qi et al., 2016; Kim C. et al., 2018; Park et al., 2018). In most of the studies, 1–200 μM (0.3–53.7 μg/ml) of concentration of formononetin was tested, and a variable response has been observed on different cell lines (Tay et al., 2019). Coumestrol is also an important anticancer isoflavonoid candidate molecule with an IC_50_ value of 228 nM tested against casein kinase 2 (CK2) (Liu S. et al., 2013). Selective reduction in CK2 activity has been seen for coumestrol in a dose-dependent manner in various cancer cell lines (Liu S. et al., 2013; Park et al., 2015; Kim et al., 2017). *In silico* modeling has suggested that coumestrol binds to ATP binding pocket of haspin kinase to suppress its activity and results in inhibition of cancer cell proliferation (Kim et al., 2017).

## Isoflavonoid Chemistry, Biosynthesis and Regulation

### Isoflavonoid Chemistry

Isoflavonoids are a diverse and distinctive subclass of flavonoids, and despite their limited distribution in the plant kingdom, isoflavonoids are structurally very diverse (Stobiecki and Kachlicki, 2006; Figure 1). The number and complexity of substitution on the basic 3-phenylchroman skeleton along with the different levels of oxidation and the presence of additional heterocyclic rings are responsible for such an outstanding diversity. Isoflavonoids are further divided into several groups, which are shown in Figure 1 (Dixon and Steele, 1999).

The ring system of isoflavones is derived from two different pathways: the A-ring is derived from the acetate pathway, whereas the B- and C-rings are formed from the shikimate pathway, resulting in a basic C6-C3-C6 skeleton (Ververidis et al., 2007). The basic skeleton is then further decorated with various rounds of glycosylation, methylation and hydroxylation reactions performed by several enzymes and enzyme complexes. This versatile decoration of the basic isoflavonoid skeleton is responsible for the enormous diversity of isoflavonoids. In general, the most common hydroxylation sites of isoflavonoids are 5,7,2′,3′-, and 4′-C, and common C- and O-glycosylation and methylation sites are 6,7, 8-, and 4′-C (Stobiecki and Kachlicki, 2006). Naturally, isoflavonoids are present in a glycosylated form in a plant cell, and the dominant glycosidic form is β-D-glycoside. Other glycosylated forms like 6 ″-*O*-acetyl-glycoside and 6 ″-*O*-malonyl-glycoside are also possible; however, the aglycone form is sometimes biologically more active (Ko, 2014). Pterocarpans are an interesting group of isoflavonoids due to the presence of the fourth ring, which is formed due to fusion of 4-C keto group with 6′-C; and due to this fusion, the ring system of pterocarpans is renamed (Whitten et al., 1997).

### Isoflavonoid Biosynthesis in Plants

The isoflavonoids are synthesized via the phenylpropanoid pathway, utilizing flavonoid intermediates. First, the aromatic amino acids (phenylalanine) are transformed into the *p*-coumaroyl CoA by a set of three enzymes: phenylalanine ammonia lyase (PAL), *trans*-cinnamate-4-hydroxylase (C4H) and 4-coumaroyl CoA lyase (4CL). Some plant species have a promiscuous PAL, which is also able to incorporate tyrosine in the pathway (Rosler et al., 1997). The next set of three important enzymes—chalcone synthase (CHS), chalcone isomerase (CHI), and chalcone reductase (CHR)—is responsible for producing naringenin and liquiritigenin from *p*-coumaroyl CoA. A detailed overview of flavonoid biosynthesis has been recently published, and readers are requested to consult Nabavi et al. (2020) and references therein for further details. Both naringenin and liquiritigenin are important flavanones, which are intermediates for various other flavonoid subgroups such as anthocyanins, proanthocyanidins, flavonols, and flavones as well as precursors for isoflavonoids.

**FIGURE 4:**
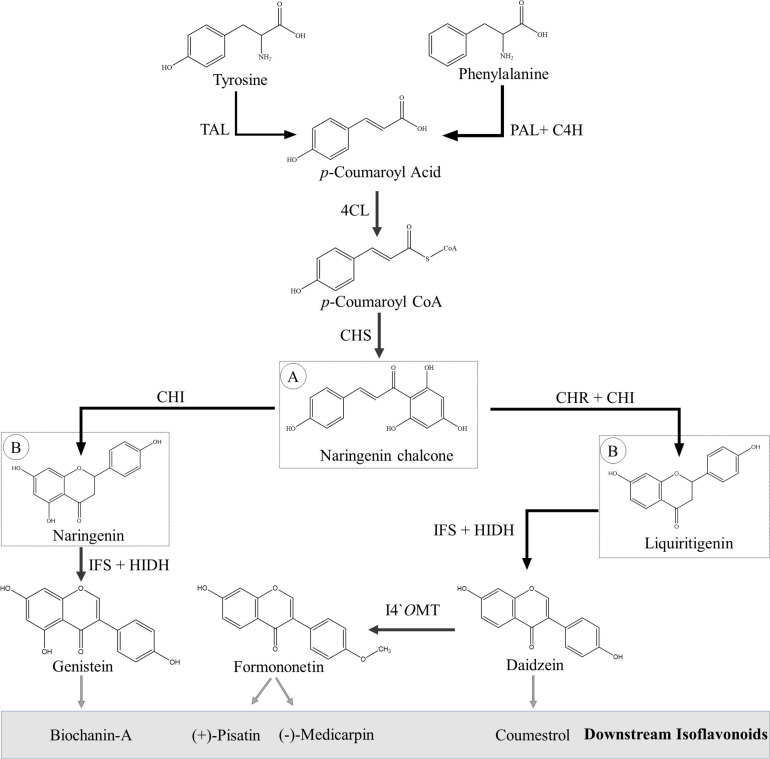
Biosynthesis of basic isoflavonoids from amino acids (phenylalanine and tyrosine). **(A)** Flavonoid precursor molecule. **(B)** Isoflavonoid precursor molecules. Abbreviations, 4CL, 4-coumarate CoA ligase; C4H, *trans*-cinnamate 4-hydroxylase; CHI, chalcone isomerase; CHR, chalcone reductase; CHS, chalcone synthase; HIDH, 2-hydroxyisoflavanone dehydratase; I4′OMT, isoflavone 4′-*O*-methyltransferase; IFS, isoflavone synthase; PAL, phenylalanine ammonia lyase; TAL, tyrosine ammonia lyase.

Leguminous plants produce isoflavonoids via two different routes, which, however, share many of their chemical reactions and biogenetic machinery. Migration of B-ring from the C-2 position to C-3 position is the first committed and unique step in isoflavonoid biosynthesis, which is catalyzed by isoflavone synthase (IFS), a cytochrome P450 class CYP93C enzyme (Steele et al., 1999; Jung et al., 2000; Supplementary Table 1). The immediate product of this reaction is 2,7,4′-trihydroxyisoflavanone, which is an unstable compound and dehydrated to corresponding isoflavanone, i.e., genistein or daidzein, either spontaneously or with the action of another enzyme, 2-hydroxyisoflavanone dehydratase (HIDH) (Steele et al., 1999; Akashi et al., 2005). Daidzein, formononetin, genistein, biochanin-A (isoflavones), pisatin, medicarpin (pterocarpans), and coumestrol (coumestans) are key isoflavonoids that are well-known for their potential pharmaceutical applications (Du et al., 2010). Biosynthesis of key isoflavonoids is discussed in detail in the following paragraphs and shown in Figure 4 and Supplementary Figures 1–4.

Formononetin is an important isoflavone that is synthesized from daidzein is a single-step reaction catalyzed by isoflavanone 4′-*O*-methyltransferase (I4′OMT). I4′OMT, first identified from *Medicago truncatula*, transfer a methyl group from *S*-adenosyl-L-methionine (SAM) to the 4′-C position of daidzein (Liu et al., 2006). Other carbons of the basic skeleton can also be methylated; for example, in alfalfa, 7-C is methylated to produce isoformononetin by isoflavone-7-*O*-methyltransferase (I7OMT) (Zubieta et al., 2001). Sometimes, both 4′-C and 7-C sites are methylated, and the resulting product is known as dimethyldaidzein (Preedy, 2012).

Pisatin is an important phytoalexin that belongs to the pterocarpan group of isoflavonoids; and like coumestans, these compounds have two asymmetric carbons, C-6a and C-11a (Slade et al., 2005). Only dextrorotatory pterocarpans [(+)-pterocarpans] possess the antimicrobial activity and are produced in a few plant species such as peanut (*Arachis hypogaea*) (Strange et al., 1985). Pisatin is the first chemically identified (+)-pterocarpan that is exclusively synthesized by pea (*P. sativum*) (Cruickshank and Perrin, 1960). Starting from formononetin, the first chemical reaction in (+)-pisatin biosynthesis pathway is catalyzed by isoflavone 3′-hydroxylase (I3′H), a P450 class CYP81E9 enzyme that adds OH group at 3′-C of formononetin to form calycosin (Supplementary Figure 1). Calycosin is then converted to pseudobaptigenin by the action of pseudobaptigenin synthase (PBS) (Clemens and Barz, 1996; Liu et al., 2003). The next step is the formation of 2′,7-dihydroxy-4′,5′-methylenedioxyisoflavone (DMD) by another P450 class CYP81E1/E7 enzyme known as isoflavone 2′-hydroxylase (I2′H) (Akashi et al., 1998; Liu et al., 2003). The next chemical reaction is catalyzed by isoflavone reductase (IFR), a unique enzyme of the pathway that introduces chirality in pterocarpan biosynthesis that converts DMD to (3*R*)-sophorol (Tiemann et al., 1987; Uchida et al., 2017). The next step to IFR is the formation of (3*R*,4*R*)-2′-hydroxyisoflavanol from (3*R*)-sophorol by the help of 2′-hydroxyisoflavanone 4-reductase (I4′R). Pea I4′R is also known as sophorol reductase (SOR), as it specifically converts (3*R*)-sophorol to (3*R*,4*R*)-7,2′-dihydroxy-4′5′-methylenedioxyisoflavanol [*cis*-(−)-DMDI] (DiCenzo and VanEtten, 2006). RNA-mediated downregulation of IFS and SOR genes in pea resulted in decreased accumulation of (+)-pisatin, which indicates that (+)-pisatin synthesis proceeds through (3*R*)-sophorol and *cis*-(−)-DMDI intermediates (Kaimoyo and VanEtten, 2008). The next step to SOR is the catalyzation of isoflav-3-enes synthase (I3S), identified very recently, which converts *cis*-(−)-DMDI to 7,2′-dihydroxy-4′,5′-methylenedioxyisoflav-3-ene (DMDIF) (Uchida et al., 2020). The enzyme involved in the next step, conversion of achiral DMDIF to (+)-6*a*-hydroxymaackiain (+)-6*a*-(HMK), is not yet identified; however, it is believed that (+)-6*a*-(HMK) is the direct precursor of (+)-pisatin. The final methylation reaction is catalyzed by (+)-6*a*-hydroxymaackiain 3-*O*-methyltransferase (HMM), which converts (+)-6*a*-(HMK) to (+)-pisatin (Wu et al., 1997).

Medicarpin is another important pterocarpan that is formed from formononetin (Supplementary Figure 2). I2′H and IFR catalyze the initial chemical reaction in medicarpin biosynthesis (Paiva et al., 1991). I2′H performs oxidation reaction at 2′-C and produces 2′-hydroxy formononetin, which is then reduced to (−)-vestitone by IFR (Tiemann et al., 1987). Two reactions are catalyzed by vestitone reductase (VR) and 7,2′-dihydroxy-4′-methoxyisoflavanol dehydratase (DMID); and (−)-vestitone is reduced and dehydrated to (−)-medicarpin, a major phytoalexin of alfalfa (*Medicago sativa*) (Guo et al., 1994).

Coumestrol belongs to the coumestans group of isoflavonoid; and like pterocarpans, these compounds also have two asymmetric carbons, C-6a and C-11a; however, only *cis*-configurations are sterically possible and present in nature (Whitten et al., 1997). The biosynthetic pathway of coumestrol synthesis is not completely understood; however, few steps have been genetically tested, and the remaining are predicted based on differential gene expression and clustering analysis (Ha et al., 2019; Supplementary Figure 3). The pathway starts from daidzein, and the first two chemical reactions involve the conversion of daidzein to 2′-hydroxydaidzein to (3*R*)-2′-hydroxydihydrodaidzein catalyzed by I2′H and IFR, respectively (Dewick et al., 1970; Berlin et al., 1972). Dehydration of (3*R*)-2′-hydroxydihydrodaidzein to 3,9-dihydroxyterocarp-6*a*-en and following chemical reactions up to coumestrol biosynthesis are NAD(P)-dependent redox reactions catalyzed by unidentified NAD(P)-linked oxidoreductases. Overall, seven genes are predicted to be involved in coumestrol biosynthesis starting from daidzein (Ha et al., 2019). These genes are supposed to encode NAD(P)-linked oxidoreductases, which are responsible for catalyzing NAD(P)-dependent oxidation reactions for coumestrol biosynthesis (Ha et al., 2019).

Genistein and biochanin-A (isoflavones) are also well-known isoflavonoids and are synthesized through the same pathway that starts from naringenin (second route of isoflavonoid biosynthesis) (Figure 4 and Supplementary Figure 4). IFS is responsible for the migration of B-ring from the C-2 position to C-3 position, as mentioned earlier; and the resulting product is 2-hydroxy-2,3-dihydrogenistein (Jung et al., 2000). Like 2,7,4′-trihydroxyisoflavanone, 2-hydroxygenistein is an unstable compound and dehydrated to genistein either spontaneously or with the action of another enzyme, HIDH (Akashi et al., 2005). Genistein is then methylated at the 4′-C position by I4′*O*MT, and biochanin-A is formed (Liu et al., 2006).

### Regulation of Isoflavonoid Biosynthesis in Plants

The isoflavonoid biosynthesis pathways are exceptionally complicated, and the overall accumulation of isoflavonoids inside a plant cell depends not only on pathway-specific enzymes but also on their interaction with other enzymes (Burbulis and Winkel-Shirley, 1999). Genes involved in isoflavone synthesis have shown a functional differentiation, which is because of two recent genome-duplication events: a soybean lineage-specific duplication (13 million years ago) and an early legume duplication (59 million years ago) (Schmutz et al., 2010; Chu et al., 2014). In the following paragraph, several factors that influence isoflavonoid biosynthesis in the host plant and the underlying molecular mechanisms are discussed.

Abiotic and biotic factors are key regulators of isoflavonoid pathway genes because anything that upregulated the expression of CHS and IFS will have a strong effect on the overall synthesis and accumulation (Dhaubhadel et al., 2003, 2007; Cheng et al., 2008). This is so because CHS and IFS are important enzymes in isoflavonoid biosynthesis, as the farmer directs the C-flow from the phenylpropanoid pathway to flavonoids, and the latter diverts C-flow from the flavonoid pathway to isoflavonoids. In one study, the authors have reported that the accumulation of isoflavonoids and the expression of four key genes (CHS7, CHS8, IFS1, and IFS2) were increased in soybean plants under high temperature stress (Chennupati et al., 2012). However, scientific evidence to explain a direct positive correlation between temperature, gene expression and accumulation of isoflavonoid content is very few. The correlation between the expression of CHS and IFS genes, isoflavonoid accumulation and effect of biotic and abiotic stresses in soybean has also been investigated (Chen et al., 2009; Devi et al., 2020). In biotic factors, *Aspergillus niger* has been tested as an elicitor of isoflavonoid biosynthesis at 0.1% concentration and a nearly fourfold increase in *IFS1* expression, and 5.9-fold increase in isoflavonoid accumulation has been documented as compared with control plants (Devi et al., 2020). Recently, Murakami and colleagues have demonstrated that the content of daidzein and formononetin is increased following the herbivory of *Spodoptera litura* and foliar applications of *S. litura* oral sections (Murakami et al., 2014).

The effect of phytohormones as elicitors for isoflavonoid biosynthesis has also been tested, as plant hormones are extensively studied as signaling molecules involved in defense response to environmental signals in plants (Creelman and Mullet, 1997; Draper, 1997). In one study, effect of salicylic acid (SA) and methyl jasmonate (MJ) was analyzed, and it was noted that SA was more active than MJ. A fivefold increase in the expression of *IFS2*, onefold increase in the expression of *CHS8* and a 4.5-fold increase in isoflavonoid contents were observed as compared with control when SA was applied at the concentration of 10 μM. Similarly, when MJ was applied at the same concentration, a maximum of onefold increase in the expression of *IFS1* and 3.75-fold increase in isoflavonoid accumulation were seen over control (Devi et al., 2020). In another recent study, an overall correlation between abscisic acid (ABA) and UV-B-induced isoflavonoid accumulation and its molecular basis have been investigated. ABA along with guanosine-3′,5′-cyclic monophosphate (cGMP) can upregulate the expression of *CHS* and *IFS* genes and ultimately result in higher accumulation of isoflavonoids under UV-B treatment in soybean. ABA causes inhibition of type 2C protein phosphate (PP2C) and activation of SNF1-related protein kinase (SnRK) and upregulate the expression of *CHS* and *IFS* and finally results in higher accumulation of isoflavonoids in the plant cell (Jiao and Gu, 2019). Overall, the role of the IFS enzyme is more critical than that of CHS, as plants always try to accumulate transcript of *IFS* in higher concentrations than *CHS*. Therefore, it is speculated that either the IFS enzyme has translational regulation or it is enzymatically very slow and has a short lifespan.

Some transcription factors (TFs) of the MYB class involved in isoflavonoid biosynthesis have been identified. The first interesting candidate is R1-type MYB TF GmMYB176, which regulates the expression of *CHS8* and thus controls the overall flavonoid/isoflavonoid contents in plants (Yi et al., 2010). Similarly, the R2R3-type MYB TFs GmMYB39 and GmMYB100 are reported to downregulate the expression of structural genes of the isoflavonoids pathway, thus negatively controlling isoflavonoid biosynthesis in plants. Recently, a soybean TF, GmMYB29, has been characterized for its positive role in isoflavonoid biosynthesis. Comparative genomic analyses have shown that GmMYB29 has maintained the highly conserved R2R3 domain and small amino acid motif in the C-terminal region, which is related to stress resistance in plants (Chu et al., 2017). The expression pattern of GmMYB29 is similar to that of *IFS2*, which supports the hypothesis that the GmMYB29 is a regulator of the *IFS2* gene (Höll et al., 2013). A positive correlation between the expression of GmMYB29 and the accumulation of isoflavonoids in different tissues of plants has also been documented (Liu X. et al., 2013; Yan et al., 2015). It is said that the soybean genome has 4,343 predicted TF encoding genes, which are roughly equal to 6.5% of the total number of genes in the plant (Doerge, 2002). Therefore, it seems that more TF involved in isoflavonoid biosynthesis and regulation will be identified and characterized in the future.

Gene expression is also regulated at the post-transcriptional level, which is largely mediated by two small RNA classes: microRNA (miRNA) and short interfering RNA (siRNA) (Khraiwesh et al., 2010). SiRNA-based regulation of the flavonoid pathway was first reported by Tuteja et al. (2009). The authors have reported tissue-specific (seed coat) silencing of the *CHS* by siRNA in soybean. In *Arabidopsis*, miRNAs like miR156, miR163, miR393 and miR828 are reported to be involved in the regulation of synthesis of secondary metabolites (Bulgakov and Avramenko, 2015; Gupta et al., 2017a). Additionally, miR156-SPL (Squamosa Promoter Binding Protein like) target pair destabilizes WD40-bHLH-MYB TF and negatively regulates anthocyanin biosynthesis in plants (Gou et al., 2011). The role of miRNA in the secondary metabolism of several medicinal plants like *Catharanthus roseus*, *Papaver somniferum*, and *Picrorhiza kurroa* have also been reported (Hao et al., 2012; Pani and Mahapatra, 2013; Boke et al., 2015; Vashisht et al., 2015). As most of the isoflavone biosynthesis and accumulation occur in the developing seed, five new miRNA and their target genes that were predicted to be involved in isoflavonoid biosynthesis have been identified (Gupta et al., 2017b). Interestingly, expression correlation analysis of Gma-miRNA26/28 and their corresponding targets 4CL and I7′*O*MT genes have shown a perfect negative correlation across all stages and genotypes studied so far. It means that decreasing the expression of Gma-miRNA26 has resulted in increase of the expression of 4CL, which could potentially divert the flux toward the synthesis of phenylpropanoid pathways and also resulted in increased accumulation of total isoflavone in the respective plant (Gupta et al., 2017b).

## Heterologous Biosynthesis of Isoflavonoids

Isoflavonoids are naturally mostly produced in legumes or pea family; however, bulk production of isoflavonoid faces some challenges due to their low content in parent plants. Therefore, alternative cost-effective production platforms are required to meet the growing demand and to ensure availability throughout the year. Model plants like *Nicotiana benthamiana* are considered useful transient expression hosts, as necessary cofactors and substrate pool are likely to be maintained *in planta* (Cravens et al., 2019). However, genetic manipulations, even for model plants, are difficult and slow as compared with microorganisms, and thus, microbial hosts are usually preferred.

Microorganisms are excellent production hosts for plant natural products (PNPs) due to low genetic complexity, ease in genetic manipulation, availability of genetic tool kit and genetic tractability. During the last couple of decades, researchers have engineered artificial isoflavonoid biosynthesis pathways in *Saccharomyces cerevisiae* and *Escherichia coli* (Cress et al., 2013; Table 2). At least seven enzymes (PAL/TAL, 4CL, CHS, CHI, CHR, IFS, and IFD) are required for the *de novo* synthesis of parent isoflavonoids: daidzein and genistein. The expression and functionality of plant P450 class enzymes are not optimal in heterologous hosts; therefore, the heterologous synthesis of isoflavonoids is challenging.

**TABLE 2:** Isoflavonoid biosynthesis using heterologous hosts (microorganisms).

Compound	Host strains	Precursors	Titer or productivity	Heterologous enzymes	Approaches	References
Daidzein	*Escherichia coli*	Liquiritigenin	18 mg/g CDW	1	Enzyme engineering for functional expression of IFS in prokaryotic system	Leonard and Koffas, 2007
	*Saccharomyces cerevisiae*	Liquiritigenin	n.e.	1	Functional analysis of IFS in yeast microsome-based system	Akashi et al., 1999
Daidzin	*S. cerevisiae*	Daidzein	n.e.	1	*In vivo* functional analysis of UDP-glycosyltransferases (UGT)	Li et al., 2014
Ononin	*S. cerevisiae*	Formononetin	n.e.	1	*In vivo* functional analysis of UDP-glycosyltransferases (UGT)	Li et al., 2014
4′−*O*−Methyl daidzein	*E. coli*	Daidzein	102.88 mg/L	2	Methylation of parent compound to improve absorption and bioavailability	Koirala et al., 2019
Daidzein-7-*O*-phosphate	*Bacillus amyloliquefaciens*	Daidzein	n.e.	−	Screening of hosts for efficient biotransformation of daidzein	Kim K.-M. et al., 2018
3′-Hydroxydaidzein	*E. coli*	Daidzein	75 mg/L	1	Screening for candidate enzyme for regioselective hydroxylation	Lee et al., 2014
Genistein	*S. cerevisiae*	Naringenin	n.e.	1	Functional analysis of IFS in yeast microsome-based system	Akashi et al., 1999
	*S. cerevisiae*	Naringenin	0.87 mg/L	2	Functional expression of IFS and CPR in yeast	Kim et al., 2005
	*E. coli*	Naringenin	10 mg/g CDW	1	Enzyme engineering for functional expression of IFS in prokaryotic system	Leonard and Koffas, 2007
	*E. coli*/*S. cerevisiae*	Tyrosine	6 mg/L	5	Co-culturing to achieve higher titer and to manage IFS expression (in yeast)	Katsuyama et al., 2007
	*S. cerevisiae*	*p*-Coumaroyl *N*-acetylcysteamine	340 μg/L	3	Synthesis of isoflavonoids from modified precursors	Katsuyama et al., 2007
	*S. cerevisiae*	Phenylalanine	0.1 mg/L	7	Construction of complete pathway for *de novo* synthesis of (iso)flavonoids	Trantas et al., 2009
	*E. coli*	Naringenin	0.67 mg/L	1	Enzyme engineering for functional expression of IFS in prokaryotic system	Kim et al., 2009
	*E. coli*	Naringenin	35 mg/L	1	Enzyme engineering for functional expression of IFS in prokaryotic system	Kim, 2020
	*E. coli*	*p*-Coumaric acid	18.6 mg/L	4	Enzyme engineering for functional expression of IFS in prokaryotic system	Kim, 2020
4′−*O*−Methyl genistein	*E. coli*	Genistein	46.81 mg/L	2	Methylation of parent compound to improve absorption and bioavailability	Koirala et al., 2019

*CDW, cell dry weight; n.e., not estimated.*

### Engineering *Saccharomyces cerevisiae* for Isoflavonoid Production

Among the many production chassis available, *S. cerevisiae* is most commonly used for heterologous PNP synthesis, as being eukaryote, it has most of the cellular compartments found in the plant cell. Availability of genetic tool kit, high rate of genetic recombination, ease in genomic manipulation and integration along with generally recognised as safe (GRAS) status are some of the additional benefits for using yeast as a heterologous host (Cravens et al., 2019).

Initial attempts to synthesize isoflavonoids in yeast were focused to convert a flavonoid precursor into the corresponding isoflavonoid. Akashi et al. were the first to express the *IFS* gene from licorice in yeast and have successfully synthesized genistein from naringenin (Akashi et al., 1999). Kim et al. have reported that an engineered yeast strain can produce up to 20 mg/L of genistein when the necessary IFS/CPR is expressed and naringenin is added in the medium (Kim et al., 2005). *De novo* synthesis (construction of the complete pathway in the engineered microbial host) of isoflavonoids has also been achieved (Trantas et al., 2009; Rodriguez et al., 2017). An engineered yeast strain, overexpressing seven heterologous enzymes, was able to produce genistein from different precursors added in the growth medium. The final yield of genistein was 7.7 mg/L (28.5 μM), 0.14 mg/L (0.52 μM), and 0.1 mM (0.4 μM) when 0.5 mM of naringenin, 1 mM of *p*-coumaric acid and 10 mM of phenylalanine were added in the media, respectively (Trantas et al., 2009). An interesting case was the synthesis of quercetin, which was not detected in the medium when phenylalanine was used as a precursor (eight heterologous enzyme reactions), but the engineered strain was able to synthesize 0.26 mg/L (0.9 μM) of quercetin when *p*-coumaric acid was added in the medium (six heterologous enzyme reactions) (Trantas et al., 2009). Recently, with further advancement in knowledge, the synthesis of quercetin has been achieved (eight heterologous enzyme reactions); however, it is speculated that *de novo* synthesis of other isoflavonoids is difficult and requires multiple rounds of genetic engineering (Rodriguez et al., 2017). Employing yeast as a production host for the heterologous synthesis of isoflavonoids has distinctive advantages in the functional expression of plant P450 class enzymes; however, further knowledge and optimization are still needed for the synthesis of key isoflavonoids.

### Engineering *Escherichia coli* for Isoflavonoid Production

*E. coli* has also been extensively used for heterologous biosynthesis of natural products due to the availability of genetic tools, ease in the engineering of native biochemical pathways, simple cultivation techniques and rapid growth (Yang et al., 2020). However, *E. coli* was not the first choice of genetic engineers for the heterologous biosynthesis of isoflavonoids due to issues in the expression of IFS and other plant P450 class enzymes.

The functional expression of IFS was the first bottleneck for the synthesis of isoflavonoids in prokaryotic hosts; therefore, efforts were put forward to express plant P450 enzymes in *E. coli*. Kim et al. (2009) were the first to engineer IFS of red clover by in-frame fusion with a CPR from rice, and the resulting chimeric protein was able to synthesize genistein (up to 15.1 mg/L) from naringenin. In the same year, Leonard et al. have also expressed an engineered chimeric IFS in *E. coli* and have successfully converted naringenin and liquiritigenin into genistein and daidzein, respectively. The *IFS* gene from *Glycine max* and CPR from *C. roseus* were used, and the membrane-spanning regions were replaced with a mammalian leader sequence (Leonard et al., 2009). Co-cultivation of *S. cerevisiae* and *E. coli* strategy was also exploited to boost the final yield of the desired isoflavonoid. In one study, when a naringenin-producing *E. coli* strain was co-cultured with *IFS* expressing *S. cerevisiae* strain, up to 6 mg/L of genistein was detected in the medium (Katsuyama et al., 2007). In recent years, significant progress has been made in heterologous product synthesis, and many PNPs are now being produced and commercialized. Some of these developments are discussed in section “Advance Genetic Engineering Approaches for *ex-planta* Isoflavonoids Biosynthesis.”

## Advanced Genetic Engineering Approaches for *Ex-planta* Isoflavonoid Biosynthesis

Significant advancement in biotechnology and synthetic biology has been made during the last couple of decades, and heterologous biosynthesis of many PNPs has been successfully achieved. As discussed in section “Heterologous Biosynthesis of Isoflavonoids,” many attempts have also been made to synthesize isoflavonoids in the microbial host, as heterologous synthesis has several advantages over natural and organic synthesis. Some of these are cost-effectiveness, environmentally friendly, on-demand production and being easy to operate, and it is also a desired technology in the green economy initiative. Therefore, in the following section, some advancements in synthetic biology and metabolic engineering are discussed, which will be helpful to produce industrially acceptable titre, rate and yield (TRY) of heterologous synthesis of most of the key isoflavonoids.

### Enzyme Engineering Approaches

Most of the enzymes involved in the synthesis of isoflavonoids are yet to be characterized; thus, identification and characterization of specific and highly efficient enzymes are a key requirement for the successful heterologous biosynthesis of isoflavonoids. Increasing availability of genetic information is helping enzyme identification and characterization efforts, and genome sequencing projects will speed up the process (One Thousand Plant Transcriptomes Initiative, 2019). If genome sequences are available, it becomes very easy to identify or discover new enzymes by comparing plants that produce or lack a specific compound, which will then help us to explain which enzyme might be the candidate for the biosynthesis of that compound. Following the gene identification, approaches like codon optimization, promoter and other regulatory sequence selections and, finally, protein engineering can be used to improve the enzyme activity and expression level (Lee et al., 2016). Therefore, engineering of the heterologous host that can produce an isoflavonoid of interest at industrially acceptable yield is not easy and straightforward.

As mentioned in Subsection “Isoflavonoid biosynthesis in plants,” enzymes involved in the synthesis of key isoflavonoids are identified but not well characterized, and suboptimal expression of an enzyme results in bottlenecks in a diversion of C-flux from central metabolism to a final product (Supplementary Table 1). Enzyme engineering can help to address some of the issues of low expression and/or low activity of plant-origin enzymes in a heterologous host such as issues of incorrect folding, feedback inhibition and suboptimal pH (Cravens et al., 2019). Enzyme localization by either synthetic or RNA-based scaffolds is a powerful strategy under enzyme engineering approaches. An interesting example in this regard is the use of a synthetic scaffold to cluster a group of three enzymes of the mevalonate pathway, and 77-fold increase in the final product was observed (Dueber et al., 2009). Together with this, protein fusion can effectively reduce pathway competition by bringing the active sites of enzymes near each other and facilitate product channeling. A truncated flavonoid 3′-hydroxylase (tF3′H), a plant P450 class enzymes and a truncated P450 reductase (tCPR) were expressed together as a fusion protein, and successful synthesis of eriodictyol was achieved in *E. coli* (Zhu et al., 2014). Directed evolution is another approach that can help to increase enzyme activity, stability and substrate specificity. Directed evolution becomes even easier if the relationship between enzyme structure, sequence and function are relatively well established.

The chemical space accessed by the heterologous pathway can be expanded to a range of new products by adding additional enzymes in the pathway. In this way, it is easy to transform natural products into their halogenated, hydroxylated, methylated and glycosylated forms by using an enzyme that can accept that natural product as a substrate. Additionally, protein engineering techniques can help to engineer existing enzymes to accept the molecule of interest, as is demonstrated for halogenase enzyme (Payne et al., 2015). The possibility to synthesize novel derivatives is another interesting opportunity. Once a pathway has been established, it is easy to add, replace or remove any pathway enzyme to produce novel derivatives.

### Pathway Engineering Approaches

Techniques used to construct metabolic pathway in the heterologous host are extensively reviewed in the literature and includes methods for multigene genomic integration, gene editing and combinatorial enzyme expression (Li et al., 2019). Recent advances in genome engineering make it possible to establish heterologous pathways in previously un-cultivatable organisms. The introduction of the CRISPR/Cas9 system for genome engineering has revolutionized metabolic engineering, as the engineering efficiency of CRISPR/Cas9 sometimes reaches 100% in *E. coli* (Cho et al., 2018). CRISPR/Cas9 variants such as catalytically dead CRISPR-associated protein 9 (dCas9) are even more useful, as they are helpful to control gene expression and to divert metabolic C-flow toward the product of interest. However, approaches to engineer a metabolic pathway in a heterologous host are not yet clear and straightforward.

Modular pathway engineering approaches are helping the researchers to get a higher titer of the product of interest. This approach involves the engineering of a parent strain to increase substrate supply, improvement in the overall flux of the selected pathway and elimination or downregulation of side products. The first interesting example in this regard is the development of a yeast strain for high *p*-coumaric acid production, as it is an important intermediate in the isoflavonoid pathway. Rodriguez and colleagues have developed a yeast strain capable of producing 1.9 g/L of *p*-coumaric acid through a combination of six genetic changes in the central metabolism (Rodriguez et al., 2015). As discussed in section “Heterologous Biosynthesis of Isoflavonoids,” the expression of CHS and IFS is not optimal, and to optimize the overall flux of the pathway, researchers have concluded that the optimal copy number for CHS and IFS is 5 and 2, respectively (Lv et al., 2019). Following further optimization of pH and carbon/nitrogen ratio (C/N), the engineered strain was capable of producing 252.4 mg/L of naringenin from glucose in a shake flask culture (Lv et al., 2019). Side product formation, i.e., phloretic acid formation via reduction of *p*-coumaroyl CoA, is also a hurdle in getting a commercially acceptable yield, as it results in metabolic C loss. Researchers have finally identified the enzyme, enoyl reductase Tsc13, responsible for phloretic acid formation. However, Tsc13 is an essential enzyme and cannot be deleted. Therefore, yeast *TSC13* is replaced with a plant *TSC13*, and the unwanted side reaction is eliminated while retaining the natural function of Tsc13 as such (Lehka et al., 2017). Thus, significant progress has been undertaken to engineer a strain able to produce a commercially acceptable yield of an isoflavonoid molecule of interest.

The development of bio-foundries is considered revolutionary progress in pathway construction and validation (Chao et al., 2017). A bio-foundry is a collection of wet lab robotics and software developed to systematize the construction, assembly and testing of pathway parts in the host strain. Until now, bio-foundry-based approaches are used to construct only a short pathway (< 5 enzymes) or already validated pathways (Casini et al., 2018). It is not clear when enzyme discovery and characterization will be automated for long pathways (> 5 enzymatic pathway), as most of the approaches are custom-made up to now; however, the potential is huge, and once optimized, such automation will revolutionize metabolic engineering projects.

### Co-culture Approaches

It is now possible to transform and express over 25 genes in a single strain as demonstrated by Li et al. (2018). However, it is demonstrated that when the number of genes required for the biosynthesis of a compound is increased, the performance of a heterologous system is decreased, and strain optimization becomes difficult and laborious (Trantas et al., 2009). To address such issues, co-culture approach, in which two or more organisms expressing complementary genes are grown together, is an interesting and useful strategy.

The co-culture approach (growing two or more organisms having different modules of the same pathway together) can help metabolic engineering in multiple ways. The effectiveness of co-culturing approach is the most obvious in the strain re-optimization process, as it becomes easy to identify and manipulate the issue of heterologous expression in a strain expression in only a part/module of the complete pathway as compared with traditional monoculture approach (Jones and Koffas, 2016). In a traditional monoculture approach, an extension of an established heterologous pathway requires additional genes to be transformed and expressed in the previously engineered strain. This might destabilize the parent strain in a genetic or fermentation perspective and demands further rounds of optimization. Genetic re-optimization is laborious work, and sometimes, it becomes very difficult to regain the previously achieved fluxes (Wu et al., 2016). However, in poly-culture approaches, previous genetic optimizations are preserved on the one hand, and establishment/optimization of a new module into a new strain are easy (as compared with already engineered strain) on the other hand; and finally, few fermentation optimization steps are required to adjust the new strain in co-culture (Li et al., 2018). Together with this, co-culturing can also help to address the issue of the expression of plant P450 class enzymes in prokaryotic hosts, and in this way, the potential of prokaryotic systems can be used for the synthesis of isoflavonoids of interest. For example, a tyrosine-producing *E. coli* and naringenin producing yeast strain (using D-xylose as a C source) were co-cultured for naringenin biosynthesis. The optimized co-culture was able to produce up to 21.16 mg/L of naringenin from simple sugars, and the final titer was eightfolds higher than the monoculture of the engineered yeast strain (Zhang et al., 2017).

Detection and quantification of heterologous products in the microbial host by using genetic biosensors is an exciting area of research and advantages offered by co-culture opportunities have increased their importance (de Frias et al., 2018). For example, a naringenin-sensitive TF FdeR (from *Herbaspirillum seropedicae*) when combined with a green fluorescent protein (GFP) can serve as genetically encoded biosensor and helps in the detection and reporting of intracellular naringenin level (Siedler et al., 2014). Another interesting example is the development of the Systematic Evolution of Ligands by Exponential Enrichment (SELEX) method, which can activate the expression of downstream genes following activation by naringenin. A nearly threefold increase in the expression of reporter genes in *E. coli* strain expressing the riboswitch was noted when 200 mg/L of naringenin was added in the media (Jang et al., 2017). In another study, an RNA riboswitch-based biosensor module was used to control the growth of naringenin-responsive *E. coli* strains grown together in a co-culture system. A positive correlation was seen between the naringenin production (by one strain) and the expression of a reporter gene (in the second strain) (Xiu et al., 2017). The authors have concluded that a naringenin-responsive biosensor has helped the second strain to control metabolic burden, and it also allowed the authors to do module-specific strain optimization comparatively easily and efficiently.

## Conclusion and Future Directions

The isoflavonoids are an important group of plant secondary metabolites that play multiple significant roles in plants as well as in humans. The diversity of isoflavonoids, as well as other PNPs, is a result of an ongoing process of evolution that has generated rich and diverse enzyme sets and will continue to do so in the future. Genome sequencing projects for the identification of candidate genes and TFs will continue to produce a wealth of knowledge for isoflavonoid biosynthesis and engineering in plants.

In plants, the isoflavonoid-mediated natural plant defense mechanisms are a potential tool to address pre-harvest crop losses due to pests and diseases, along with reducing the use of toxic and expensive pesticides. Therefore, an attractive avenue for further research is to engineer beneficial isoflavonoid biosynthesis pathways into non-endogenous commercial crop plants. However, this approach has technical and social limitations, in terms of the complex interactions and interdependencies of functional genes with added challenges from the public perception and regulatory requirements for the use of genetically modified organisms.

The widespread availability of isoflavonoids for use in agricultural, nutraceutical and pharmaceutical applications is limited due to low yield in plants; therefore, alternative approaches such as *ex-planta* biosynthesis synthetic methods are being investigated. Most of the reports published so far have used *E. coli* and *S. cerevisiae* as heterologous hosts; however, interest in using other available microbial chassis is growing. Therefore, the identification and engineering of enzymes and other genetic components, as well as the exploration of new hosts, will set the direction of future research in the heterologous synthesis of isoflavonoids. Developments in few key areas mentioned in Box 1 will pave the way of successful biosynthesis of isoflavonoids in the near future.

**Box 1. Emerging developments in synthetic biology approaches for heterologous flavonoid biosynthesis.**

**Table:** 

Current challenges	Emerging developments
Characterization of flavonoid biosynthetic pathways and gene identification	**Genome sequencing projects and machine learning approaches will help in** ✓ Identification of microbial homologous of plant enzymes ✓ Annotation and characterization of genes from plant hosts
Slow enzyme kinetics	**Protein engineering and directed evolution will help in** ✓ Enhancing and optimizing enzyme activity and stability in prokaryotic systems ✓ Engineering binding epitopes for enhanced pathway partner protein interactions
Limitations in metabolic engineering	**CRISPR/Cas9-based genomic integration approaches will help in** ✓ Integration of natural product biosynthetic pathways into non-endogenous microorganisms ✓ Manipulation of microbial chassis ✓ Use of inexpensive microbial feed stock to reduce synthesis cost
	**Co-culturing approaches will help to** ✓ Manage gene expression, reduce metabolic burden and allow rapid testing of new biosynthetic pathways

The successful development of commercially scalable *ex-planta* production platforms for isoflavonoids will have the further benefit of opening exciting avenues toward the biosynthesis and exploitation of alternative PNPs. The realization of these efforts will pave the way toward future economically sustainable, socially beneficial, green-modular bioindustries.

## Author Contributions

MS, SRS, and PK conceived, designed, and wrote the initial draft of this review article. SS and PK reviewed and edited the manuscript. All authors have read and approved the contents of this manuscript.

## Conflict of Interest

The authors declare that the research was conducted in the absence of any commercial or financial relationships that could be construed as a potential conflict of interest.
