# Supplementary material for: Recent Advances in Heterologous Synthesis Paving Way for Future Green-Modular Bioindustries: A Review With Special Reference to Isoflavonoids
Source: Front Bioeng Biotechnol. 2021 Jul 1;9:673270. doi: 10.3389/fbioe.2021.673270 (PMC8282456; doi:10.3389/fbioe.2021.673270)
Supplement: Supplementary file 1 [file Data_Sheet_1.PDF]

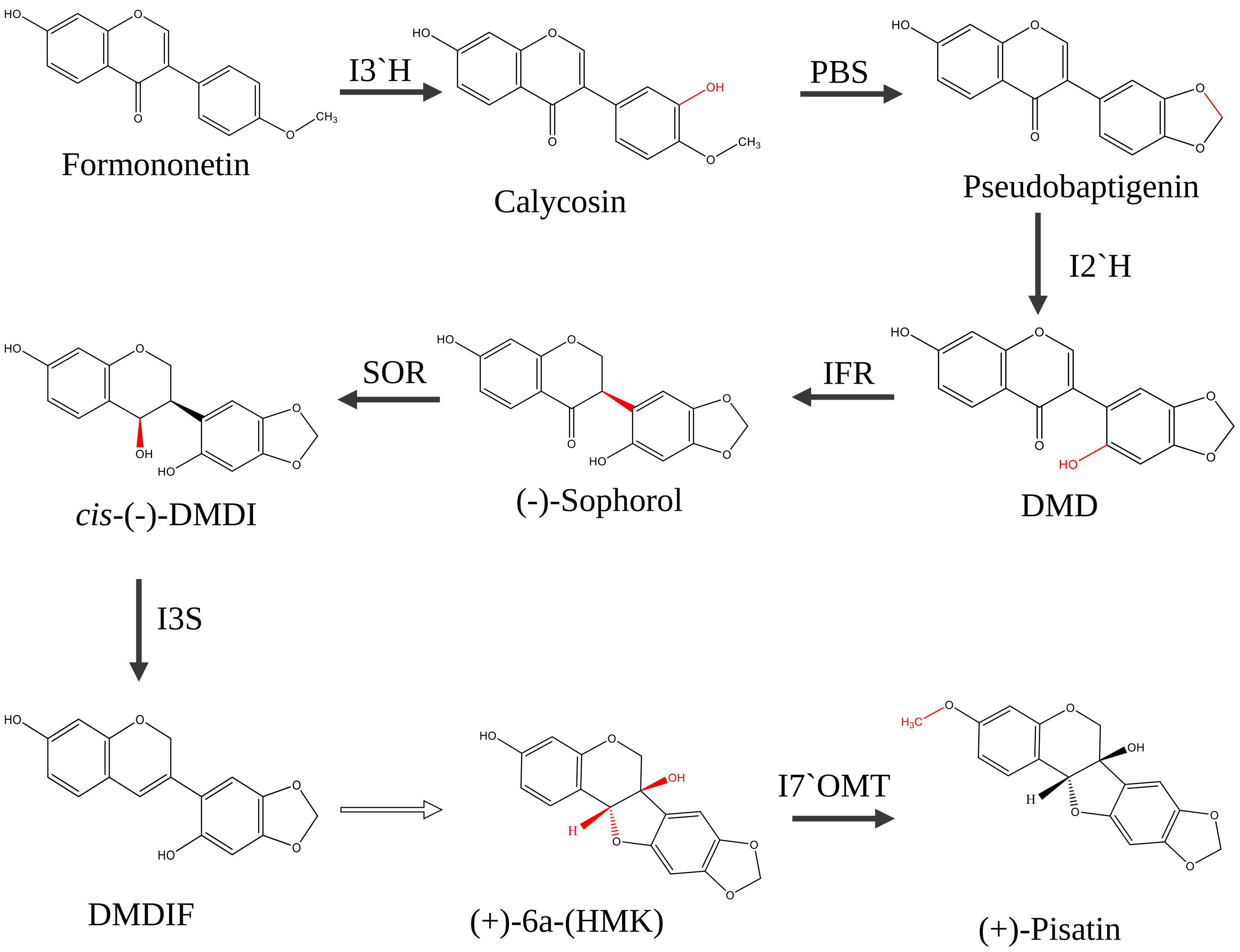

Supplementary Figure 1: Biosynthesis of Pisatin
(Empty arrow represents unidentified enzymes/steps)

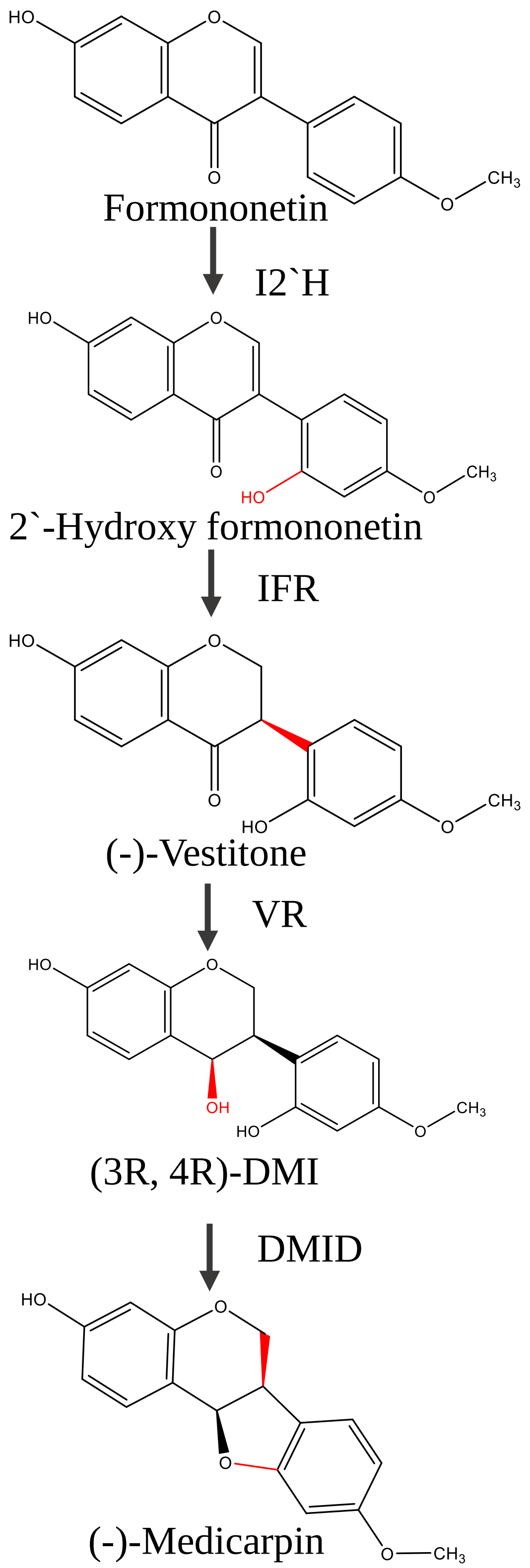

Supplementary Figure 2: Biosynthesis of Medicarpin

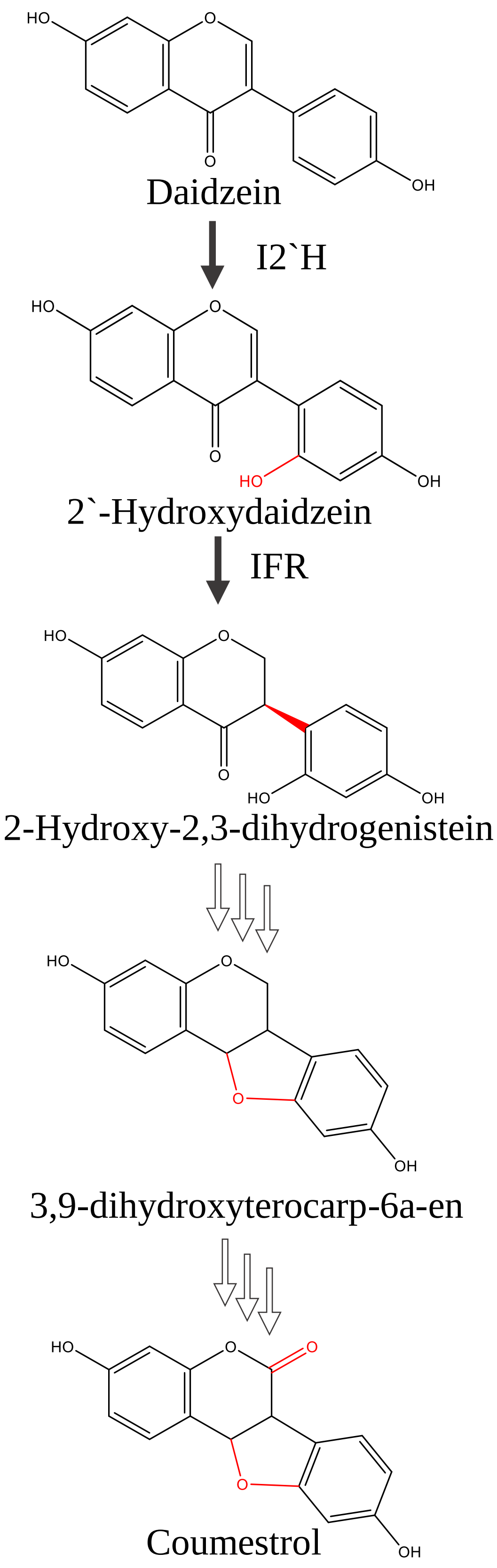

Supplementary Figure 3: Biosynthesis of Coumestrol
(Empty arrows represents multiple predicted/ unidentified enzymes/steps)

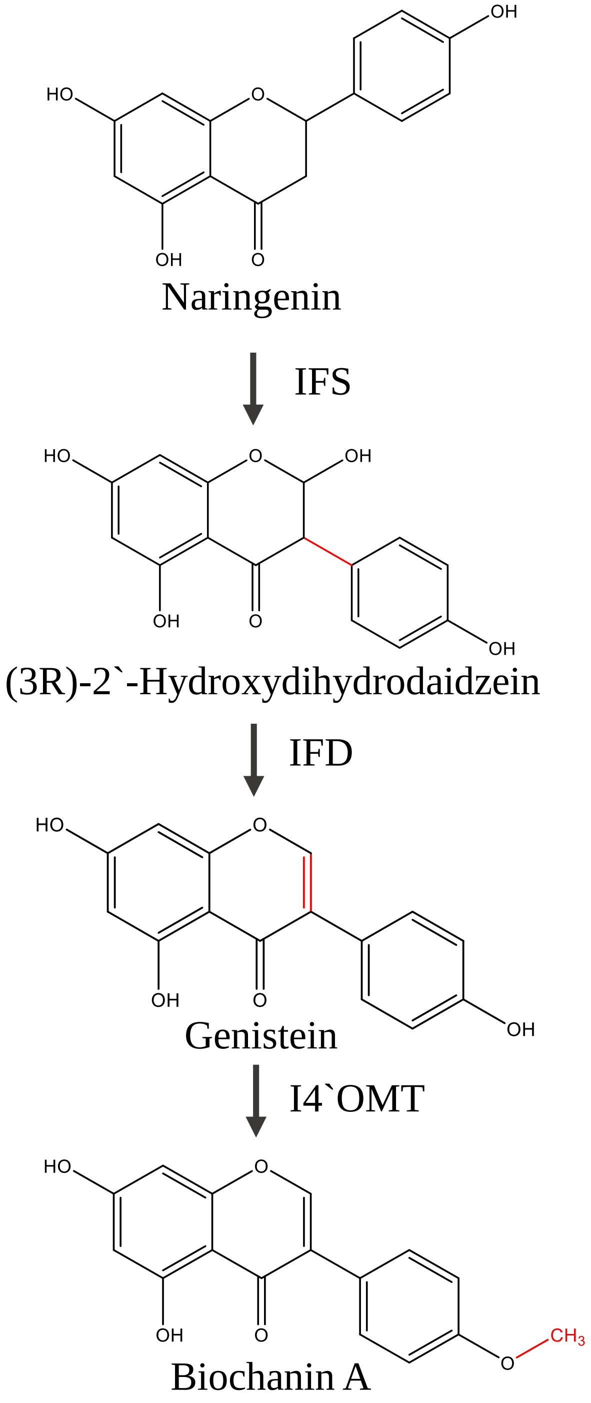

Supplementary Figure 4: Biosynthesis of Biochanin A
