# Supplementary material for: Recent Advances in Heterologous Synthesis Paving Way for Future Green-Modular Bioindustries: A Review With Special Reference to Isoflavonoids
Source: Front Bioeng Biotechnol. 2021 Jul 1;9:673270. doi: 10.3389/fbioe.2021.673270 (PMC8282456; doi:10.3389/fbioe.2021.673270)
Supplement: Supplementary file 2 [file Table_1.DOCX]

Supplementary Table 1: Enzymatic characteristics of enzymes involved in isoflavonoid biosynthesis

| Enzyme Abb. | Organism | Cofactor | Km (uM) | Kcat  (s-1) | UniProt | Reaction details and mechanism | References |
| --- | --- | --- | --- | --- | --- | --- | --- |
| PAL | *Rhodosporidium toruloides* | no | 0.29 | n.a. | P11544 | L-phenylalanine <=> trans-Cinnamate + Ammonia | (Adachi et al., 1990) |
|  |  |  |  |  |  |  |  |
|  |  | no | 0.18 | n.a | P11544 | L-tyrosine <=> p-Coumaric acid + Ammonia | (Adachi et al., 1990) |
|  |  |  |  |  |  |  |  |
| C4H | *Arabidopsis thaliana* | Heme | n.a. | n.a. | P92994 | trans-Cinnamate + [Reduced NADPH---hemoprotein reductase] + Oxygen <=> 4-Coumarate + [Oxidized NADPH---hemoprotein reductase] + H2O | (Bell-Lelong et al., 1997) |
|  |  |  |  |  |  |  |  |
| TAL | *Zea mays* | no | 19 | 0.92 | Q8VXG7 | L-tyrosine <=> p-Coumaric acid + Ammonia | (Rosler et al., 1997) |
|  |  |  |  |  |  |  |  |
|  |  | no | 270 | 10.6 | Q8VXG7 | L-phenylalanine <=> trans-Cinnamate + Ammonia | (Rosler et al., 1997) |
|  |  |  |  |  |  |  |  |
| 4CL | *Arabidopsis thaliana* | no | 23 | n.a. | Q9S777 | p-Coumaric acid + ATP + CoA <=> p-Coumaroyl-CoA + AMP + Diphosphate | (Ehlting et al., 1999) |
|  |  |  |  |  |  |  |  |

| CHS | *Medicago sativa* | no | 4.5 | n.a. | P30074 | p-Coumaryol-CoA + 3 Malonyl-CoA <=> Naringenin chalcone + 4 CoA + 3 CO_2_ | (Jez et al., 2000) |
| --- | --- | --- | --- | --- | --- | --- | --- |
|  |  |  |  |  |  |  |  |

| CHI | *Medicago sativa* | no | 112 | n.a | P28012 | Naringenin chalcone <=> Naringenin | (Jez and Noel, 2002) |
| --- | --- | --- | --- | --- | --- | --- | --- |
|  |  |  |  |  |  |  |  |
|  |  | no | 8.4 | n.a | P28012 | Isoliquiritigenin <=> Liquiritigenin | (Jez and Noel, 2002) |
|  |  |  |  |  |  |  |  |
| CHR | *Medicago sativa* | no | n.a. | n.a. |  | Naringenin chalcone <=> Isoliquiritigenin |  |
|  |  |  |  |  |  |  |  |
|  |  | no | n.a. | n.a. |  | Isoliquiritigenin <=> Liquiritigenin |  |
|  |  |  |  |  |  |  |  |

| IFS | *Glycine max* | Heme | n.a. | n.a. | Q9SWR5 | Liquiritigenin + Oxygen + [Reduced NADPH---hemoprotein reductase] <=> 2,7,4'-Trihydroxyisoflavanone + H2O + [Oxidized NADPH---hemoprotein reductase] | (Steele et al., 1999) |
| --- | --- | --- | --- | --- | --- | --- | --- |
|  |  |  |  |  |  |  |  |
|  |  |  |  |  |  | Naringenin + Oxygen + [Reduced NADPH---hemoprotein reductase] <=> 2-Hydroxy-2,3-dihydrogenistein + H2O + [Oxidized NADPH---hemoprotein reductase] |  |
|  |  |  |  |  |  |  |  |
|  |  |  |  |  |  | 6,7,4'-Trihydroxyflavanone + Oxygen + [Reduced NADPH---hemoprotein reductase] <=> 2,6,7,4'-Tetrahydroxyisoflavanone + H2O + [Oxidized NADPH---hemoprotein reductase] |  |
|  |  |  |  |  |  |  |  |

| HIDH | *Glycine max* | no | 29 | 1.6 | Q5NUF3 | 2,7-Dihydroxy-4'-methoxyisoflavanone <=> Formononetin + H2O | (Akashi et al., 2005) |
| --- | --- | --- | --- | --- | --- | --- | --- |
|  |  |  |  |  |  |  |  |
|  |  |  | 114 | 5.3 |  | 2,7,4'-Trihydroxyisoflavanone <=> Daidzein + H2O |  |
|  |  |  |  |  |  |  |  |
|  |  |  | 170 | 18.1 |  | 2-Hydroxy-2,3-dihydrogenistein <=> Genistein + H2O |  |
|  |  |  |  |  |  |  |  |

| I4`OMT | *Pisum sativum* | no | 3 | n.a. | Q84KK6 | S-Adenosyl-L-methionine + 2,7,4'-Trihydroxyisoflavanone <=> S-Adenosyl-L-homocysteine + 2,7-Dihydroxy-4'-methoxyisoflavanone | (Akashi et al., 2006) |
| --- | --- | --- | --- | --- | --- | --- | --- |
|  |  |  |  |  |  |  |  |
|  |  |  |  |  |  | Daidzein + S-Adenosyl-L-methionine <=> Formononetin + S-Adenosyl-L-homocysteine |  |
|  |  |  |  |  |  |  |  |
|  |  |  |  |  |  | S-Adenosyl-L-methionine + Genistein <=> S-Adenosyl-L-homocysteine + Biochanin A |  |
|  |  |  |  |  |  |  |  |

| I3`H | *Medicago truncatula* | Heme | 49.7 | 0.028 | Q6WNQ9 | Formononetin + [Reduced NADPH---hemoprotein reductase] + Oxygen <=> Calycosin + [Oxidized NADPH---hemoprotein reductase] + H_2_O | (Liu et al., 2003) |
| --- | --- | --- | --- | --- | --- | --- | --- |
|  |  |  |  |  |  |  |  |
|  |  |  | 112.6 | 0.1 |  | Biochanin A + [Reduced NADPH---hemoprotein reductase] + Oxygen <=> Pratensein + [Oxidized NADPH---hemoprotein reductase] + H2O |  |
|  |  |  |  |  |  |  |  |
| PBS | *Cicer arietinum* | Heme | n.a. | n.a. | n.a. | Calycosin + [Reduced NADPH---hemoprotein reductase] + Oxygen <=> Pseudobaptigenin + [Oxidized NADPH---hemoprotein reductase] + 2 H_2_O | (Clemens and Barz, 1996) |
|  |  |  |  |  |  |  |  |

| I2`H | *Medicago truncatula* | Heme | 67 | 0.015 | Q6WNR0 | Formononetin + [Reduced NADPH---hemoprotein reductase] + Oxygen <=> 2'-Hydroxyformononetin + [Oxidized NADPH---hemoprotein reductase] + H_2_O | (Liu et al., 2003) |
| --- | --- | --- | --- | --- | --- | --- | --- |
|  |  |  |  |  |  |  |  |
|  |  |  | 51 | 0.033 |  | Biochanin A + [Reduced NADPH---hemoprotein reductase] + Oxygen <=> 2'-Hydroxybiochanin A + [Oxidized NADPH---hemoprotein reductase] + H_2_O |  |
|  |  |  |  |  |  |  |  |
|  | *Glycyrrihiza enhinata* | Heme | 0 | 0 | P93147 | Pseudobaptigenin + [Reduced NADPH---hemoprotein reductase] + Oxygen <=> 2',7-Dihydroxy-4',5'-methylenedioxyisoflavone + [Oxidized NADPH---hemoprotein reductase] + H2O | (Akashi et al., 1998, 1) |
|  |  |  |  |  |  |  |  |

| HI7OMT | *Medicago sativa* | no | n.a. | n.a | O24529 | Genistein + S-Adenosyl-L-methionine <=> Prunetin + S-Adenosyl-L-homocysteine | (Zubieta et al., 2001) |
| --- | --- | --- | --- | --- | --- | --- | --- |
|  |  |  |  |  |  |  |  |
|  |  |  |  |  | O24529 | Daidzein + S-Adenosyl-L-methionine <=> Isoformononetin + S-Adenosyl-L-homocysteine |  |
|  |  |  |  |  |  |  |  |

| 2`IFR | *Medicago sativa* | no | 14.2 | n.a | P52575 | 2'-Hydroxyformononetin + NADPH + H+ <=> (-)-Vestitone + NADP+ | (Wang et al., 2006) |
| --- | --- | --- | --- | --- | --- | --- | --- |
|  |  |  |  |  |  |  |  |
|  |  |  |  |  |  | 2',7-Dihydroxy-4',5'-methylenedioxyisoflavone + NADPH + H+ <=> (-)-Sophorol + NADP+ | (Tiemann et al., 1987) |
|  |  |  |  |  |  |  |  |
|  |  |  |  |  |  | 2',7-Dihydroxy-4',5'-methylenedioxyisoflavone + NADPH + H+ <=> (+)-Sophorol + NADP+ |  |
|  |  |  |  |  |  |  |  |
| SOR | *Pisum sativum* | n.a. | n.a. | n.a. | n.a. | (-)-Sophorol <=> 7,2'-Dihydroxy-4'-methoxy-isoflavanol | (Uchida et al., 2020) |
|  |  |  |  |  |  |  |  |

| VR | Medicago sativa | no | 40 | n.a. | Q40316 | (-)-Vestitone + NADPH + H+ <=> 7,2'-Dihydroxy-4'-methoxy-isoflavanol + NADP+ | (Guo and Paiva, 1995) |
| --- | --- | --- | --- | --- | --- | --- | --- |
|  |  |  |  |  |  |  |  |
| HMM | *Pisum sativum* | no | 0.5 | n.a | P0DH60 | (+)-6a-Hydroxymaackiain + S-Adenosyl-L-methionine <=> Pisatin + S-Adenosyl-L-homocysteine | (Wu et al., 1997) |
|  |  |  |  |  |  |  |  |
|  |  | no | 23 | n.a |  | S-Adenosyl-L-methionine + 2,7,4'-Trihydroxyisoflavanone <=> S-Adenosyl-L-homocysteine + 2,4`-Dihydroxy-7'-methoxyisoflavanone |  |
|  |  |  |  |  |  |  |  |

References:

Adachi, O., Matsushita, K., Shinagawa, E., and Ameyama, M. (1990). Crystallization and properties of L-phenylalanine ammonia-lyase from Rhodosporidium toruloides. *Agricultural and biological chemistry* 54, 2839–2843.

Akashi, T., Aoki, T., and Ayabe, S. (1998). CYP81E1, a cytochrome P450 cDNA of licorice (Glycyrrhiza echinataL.), encodes Isoflavone 2′-hydroxylase. *Biochemical and biophysical research communications* 251, 67–70.

Akashi, T., Aoki, T., and Ayabe, S. (2005). Molecular and biochemical characterization of 2-hydroxyisoflavanone dehydratase. Involvement of carboxylesterase-like proteins in leguminous isoflavone biosynthesis. *Plant physiology* 137, 882–891.

Akashi, T., VanEtten, H. D., Sawada, Y., Wasmann, C. C., Uchiyama, H., and Ayabe, S. (2006). Catalytic specificity of pea O-methyltransferases suggests gene duplication for (+)-pisatin biosynthesis. *Phytochemistry* 67, 2525–2530.

Bell-Lelong, D. A., Cusumano, J. C., Meyer, K., and Chapple, C. (1997). Cinnamate-4-hydroxylase expression in Arabidopsis (regulation in response to development and the environment). *Plant Physiology* 113, 729–738.

Clemens, S., and Barz, W. (1996). Cytochrome P450-dependent methylenedioxy bridge formation in Cicer arietinum. *Phytochemistry* 41, 457–460.

Ehlting, J., Büttner, D., Wang, Q., Douglas, C. J., Somssich, I. E., and Kombrink, E. (1999). Three 4‐coumarate: coenzyme A ligases in Arabidopsis thaliana represent two evolutionarily divergent classes in angiosperms. *The plant journal* 19, 9–20.

Guo, L., and Paiva, N. L. (1995). Molecular cloning and expression of alfalfa (Medicago sativa L.) vestitone reductase, the penultimate enzyme in medicarpin biosynthesis. *Archives of biochemistry and biophysics* 320, 353–360.

Jez, J. M., Ferrer, J.-L., Bowman, M. E., Dixon, R. A., and Noel, J. P. (2000). Dissection of malonyl-coenzyme A decarboxylation from polyketide formation in the reaction mechanism of a plant polyketide synthase. *Biochemistry* 39, 890–902.

Jez, J. M., and Noel, J. P. (2002). Reaction mechanism of chalcone isomerase pH dependence, diffusion control, and product binding differences. *Journal of Biological Chemistry* 277, 1361–1369.

Liu, C.-J., Huhman, D., Sumner, L. W., and Dixon, R. A. (2003). Regiospecific hydroxylation of isoflavones by cytochrome p450 81E enzymes from Medicago truncatula. *The Plant Journal* 36, 471–484.

Rosler, J., Krekel, F., Amrhein, N., and Schmid, J. (1997). Maize phenylalanine ammonia-lyase has tyrosine ammonia-lyase activity. *Plant physiology* 113, 175–179.

Steele, C. L., Gijzen, M., Qutob, D., and Dixon, R. A. (1999). Molecular characterization of the enzyme catalyzing the aryl migration reaction of isoflavonoid biosynthesis in soybean. *Archives of Biochemistry and Biophysics* 367, 146–150.

Tiemann, K., Hinderer, W., and Barz, W. (1987). Isolation of NADPH: isoflavone oxidoreductase, a new enzyme of pterocarpan phytoalexin biosynthesis in cell suspension cultures of Cicer arietinum. *FEBS letters* 213, 324–328.

Uchida, K., Aoki, T., Suzuki, H., and Akashi, T. (2020). Molecular cloning and biochemical characterization of isoflav-3-ene synthase, a key enzyme of the biosyntheses of (+)-pisatin and coumestrol. *Plant Biotechnology*, 20.0421 a.

Wang, X., He, X., Lin, J., Shao, H., Chang, Z., and Dixon, R. A. (2006). Crystal structure of isoflavone reductase from alfalfa (Medicago sativa L.). *Journal of molecular biology* 358, 1341–1352.

Wu, Q., Preisig, C. L., and VanEtten, H. D. (1997). Isolation of the cDNAs encoding (+) 6a-hydroxymaackiain 3-O-methyltransferase, the terminal step for the synthesis of the phytoalexin pisatin in Pisum satvium. *Plant molecular biology* 35, 551–560.

Zubieta, C., He, X.-Z., Dixon, R. A., and Noel, J. P. (2001). Structures of two natural product methyltransferases reveal the basis for substrate specificity in plant O-methyltransferases. *Nature structural biology* 8, 271–279.
